# F1. GENOME-WIDE ASSOCIATION STUDIES SUGGESTED ASSOCIATION BETWEEN DGKB AND ANTIPSYCHOTIC INDUCED WEIGHT GAIN IN EUROPEANS AND AFRICAN AMERICANS

**DOI:** 10.1093/schbul/sby017.532

**Published:** 2018-04-01

**Authors:** Malgorzata Maciukiewicz, Arun Tiwari, Vanessa Goncalves, Clement Zai, Eva Brandl, Natalie Freeman, Jeffrey Lieberman, Herbert Meltzer, Christopher Laughlin, Erika Nurmi, James Kennedy, Daniel Mueller

**Affiliations:** 1 Centre for Addiction and Mental Health; 2 Centre for Addiction and Mental Health, University of Toronto; 3 Charité University Clinic; 4 Columbia University; 5 Northwestern University Feinberg School of Medicine; 6 UCLA Semel Institute for Neuroscience; 7 University of California, Los Angeles; 8 University of Toronto

## Abstract

**Background:**

Schizophrenia (SCZ) is a severe, devastating disorder with a life-time prevalence of 1% irrespective of gender or ethnic group, treated primarily with antipsychotic (AP) medications. Despite clinical efficacy of APs, they are associated with severe side effects including antipsychotic-induced weight gain (AIWG).

**Methods:**

We investigated n=201 schizophrenia or schizoaffective disorder patients of European and African American ancestry who were treated mostly with clozapine or olanzapine. Individuals were genotyped on the Infinium Omni2.5 BeadChip. We conducted genome-wide association analysis for AIWG defined primarily as the percentage of weight change from baseline. Additionally, we ran pathway, enrichment, network, and polygenic risk score analyses to investigate top genes using in silico methods.

**Results:**

In the mixed sample, we observed genome-wide significant association between the diacylglycerol kinase beta (DGKB) variant (β=0.411; p=3.15 × 10–9) and percentage of weight change. The association remained nominally significant in both Europeans (β=0.271; p=0.002) and African Americans (β=0.579; p=5.73 × 10–5) for the same risk allele. In Europeans, the top variant (β=0.406; p=1.26 × 10–6) was located upstream of the Stanniocalcin 2 (STC2) gene. Bayesian fine mapping suggested the variant nearby SNP upstream of STC2 (p=0.034; PHRED=3.691, posterior prob.=0.496) to be the most significant. We noticed no significant enrichment in metabolic pathways for SNPs, but our top genes (p<5 × 10–5) were enriched in the GWAS catalog for risk of obesity (pmixed=0.018; pEuropeans=0.015) and schizophrenia (pmixed=0.006). Top genes also interacted with known risk factors for obesity (Glucose-6-Phosphate Dehydrogenase (G6PD)) and schizophrenia (NudE Neurodevelopment Protein 1 Like 1 (NDEL1)), and are targeted by microRNAs related to schizophrenia (mir-34a) and obesity (mir-19b). Polygenic risk score analyses did not provide support for major genetic overlap between obesity-related and lipid-associated SNPs and the risk of AIWG.

**Discussion:**

Our findings suggested that a variant in DGKB is associated with the percentage of weight gain in both African Americans and Europeans.

